# Reduced mitochondrial respiration and increased calcium deposits in the EDL muscle, but not in soleus, from 12-week-old dystrophic *mdx* mice

**DOI:** 10.1038/s41598-019-38609-4

**Published:** 2019-02-13

**Authors:** Rhayanna B. Gaglianone, Anderson Teixeira Santos, Flavia Fonseca Bloise, Tania Maria Ortiga-Carvalho, Manoel Luis Costa, Thereza Quirico-Santos, Wagner Seixas da Silva, Claudia Mermelstein

**Affiliations:** 1 Instituto de Ciências Biomédicas, Universidade Federal do Rio de Janeiro, Rio de Janeiro, RJ Brazil; 2 Instituto de Bioquímica Médica Leopoldo de Meis, Universidade Federal do Rio de Janeiro, Rio de Janeiro, RJ Brazil; 3 Instituto de Biofísica Carlos Chagas Filho, Universidade Federal do Rio de Janeiro, Rio de Janeiro, RJ Brazil; 4 Instituto de Biologia, Universidade Federal Fluminense, Niterói, RJ Brazil

## Abstract

Mitochondria play an important role in providing ATP for muscle contraction. Muscle physiology is compromised in Duchenne muscular dystrophy (DMD) and several studies have shown the involvement of bioenergetics. In this work we investigated the mitochondrial physiology in fibers from fast-twitch muscle (EDL) and slow-twitch muscle (soleus) in the *mdx* mouse model for DMD and in control C57BL/10J mice. In our study, multiple mitochondrial respiratory parameters were investigated in permeabilized muscle fibers from 12-week-old animals, a critical age where muscle regeneration is observed in the *mdx* mouse. Using substrates of complex I and complex II from the electron transport chain, ADP and mitochondrial inhibitors, we found in the *mdx* EDL, but not in the *mdx* soleus, a reduction in coupled respiration suggesting that ATP synthesis is affected. In addition, the oxygen consumption after addition of complex II substrate is reduced in *mdx* EDL; the maximal consumption rate (measured in the presence of uncoupler) also seems to be reduced. Mitochondria are involved in calcium regulation and we observed, using alizarin stain, calcium deposits in *mdx* muscles but not in control muscles. Interestingly, more calcium deposits were found in *mdx* EDL than in *mdx* soleus. These data provide evidence that in 12-week-old *mdx* mice, calcium is accumulated and mitochondrial function is disturbed in the fast-twitch muscle EDL, but not in the slow-twitch muscle soleus.

## Introduction

Duchenne muscular dystrophy (DMD) is a fatal muscular disorder caused by nonsense mutations, large deletions or duplications in the dystrophin gene. DMD is characterized by progressive muscle wasting. The absence of dystrophin, a membrane-associated protein, causes disruption of the dystrophin-glycoprotein complex (DGC), which is critical for maintaining sarcolemma integrity and activity of signaling complexes and ion channels. DGC disruption induces direct calcium influx and/or abnormal cytosolic calcium homeostasis, causing membrane leakage and increased vulnerability of myofibers to necrosis^1,2^. Calcium is a key regulator of cell signaling and is the main effector of skeletal muscle contraction. The availability of cytoplasmic calcium is regulated by the uptake of calcium by both the sarcoplasmic reticulum and mitochondria. Different muscle fiber types, fast- and slow-twitch, have different mitochondrial function and calcium levels.

The *mdx* mouse with defective dystrophin expression is one of the most widely used animal models for DMD research. These animals present a mild phenotype and a less severe disease course compared to humans, which is most likely due to the high regenerative capacity of mouse muscles^3–5^. Hence, *mdx* muscles present cycles of degeneration and regeneration but allow a normal lifespan, contrasting with 75% lifespan reduction in humans^5,6^.

Muscular dystrophy in *mdx* mice shows an age-dependent disease severity^7–10^. Soon after weaning (21–28 days) *mdx* mice exhibit intense inflammatory myonecrosis, causing the release of factors that activate the proliferation of quiescent satellite cells important for muscle damage recovery at adulthood. In mature adults at 12 wks, *mdx* muscles not yet affected by senescence show mild inflammatory reaction and efficient muscular regeneration^11–13^.

During the last decade the involvement of mitochondria in DMD pathogenesis has been identified by different groups^9,10,14–19^. Mitochondria are among the first cell components to be affected in DMD and a decline in mitochondrial activity over time precedes the onset of the disease symptoms^17^. Nevertheless, in relation to the different phases of the pathology, the physiological function of mitochondria has received very little attention. In particular, mitochondrial physiology in studies of the regeneration phase of the disease was barely mentioned^9,10,18^. In addition, the studies often used a pool of different muscle samples to analyze mitochondrial physiology^14^. This is an important issue, since it is well known that one of the determining factors in the study of mitochondrial physiology is the isolation procedure, due to the small tissue mass available. The use of a pool of different muscle samples makes it difficult to relate the results to specific muscle types.

Understanding the mechanisms by which *mdx* muscles can efficiently regenerate, while human DMD muscles cannot, is of special importance in this field and can open new possibilities for DMD treatment and therapy. Therefore, it is important to assess mitochondrial respiration in skeletal muscles with distinct fiber-type specialization in *mdx* mice at 12 wks. To address this point, we used permeabilized fibers from fast-twitch *extensor digitorum longus* (EDL), and slow-twitch soleus from *mdx* mice at 12 wks. We assessed mitochondrial metabolic states such as coupled and uncoupled respiration and maximal respiration capacity by successive additions of mitochondrial substrates and inhibitors to assess the functioning of the electron transport chain through high-resolution respirometry. We also analyzed the presence of calcium deposits in these muscles (EDL and soleus) in an effort to correlate the deposits with alterations in mitochondrial function.

## Results

**Figure 1:**
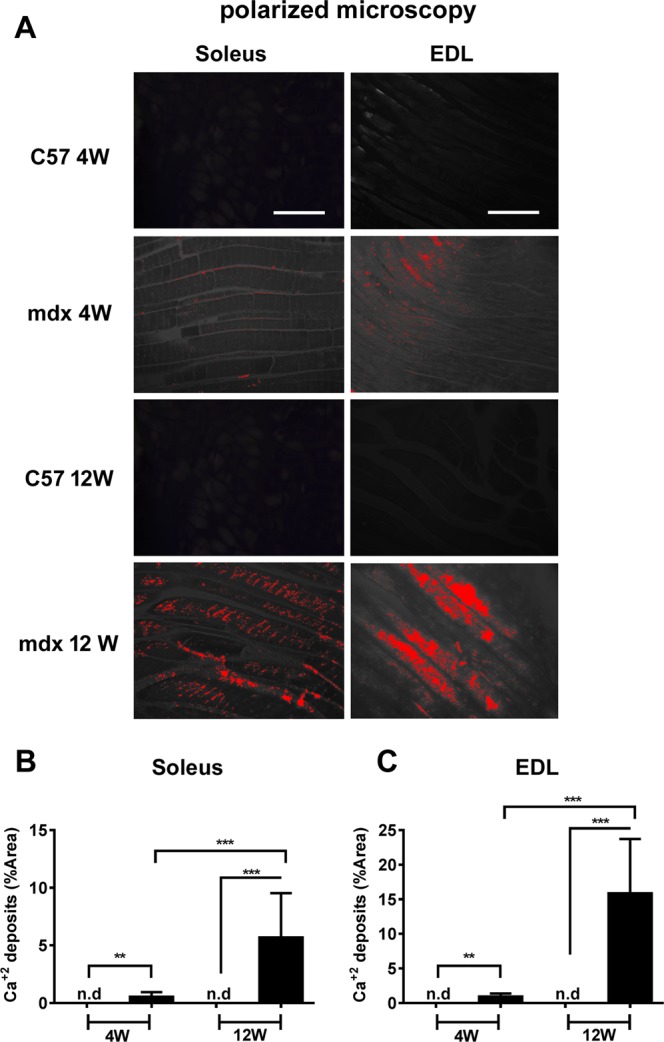
Calcium deposit quantification in soleus and EDL muscles from 4- and 12-wk-old control and *mdx* animals. Images from alizarin staining were acquired under polarized optical microscopy (**A**) and the amounts of calcium deposits were quantified in soleus (**B**) and EDL (**C**) muscles. The data are expressed as the mean ± SD of 3 animals per experimental group. (**B**) Soleus: **p = 0.0016 and ***p < 0.0001; (**C**) EDL: **p = 0.0014 and ***p < 0.0001; n.d - not detected; black bars represent *mdx*. Scale bars in soleus and in EDL images = 100 µm.

Excessive calcium influx and increased membrane permeability are early precursors of damage events in *mdx* muscular dystrophy^1,2^. Thus we decided to analyze the presence of calcium deposits in fast and slow muscles of 4- and 12-week-old *mdx* mice to try to correlate the amount of calcium in the tissue with muscle fiber type and phase of the disease. Muscle sections (EDL and soleus) were stained with Alizarin Red S (which stains calcium specifically) and images of calcium deposits were acquired under polarization microscopy and quantified (Fig. 1). Both soleus and EDL from *mdx* muscles showed detectable amounts of calcium deposits at both ages (4 and 12 wks; Fig. 1). Conversely, no calcium deposits were detected in C57 control muscles at either age (Fig. 1). Interestingly, both soleus and EDL *mdx* muscles showed an increase in calcium deposits at 12 wks in comparison with 4 wks. Importantly, we found a ~16% average increase in calcium deposits in EDL from *mdx* at 12 wks compared to EDL from control at 12 wks (Fig. 1). EDL from *mdx* at 4 wks showed ~1% increase compared to EDL from control at 4 wks, and soleus at 4 and 12 wks showed ~1% and ~6% increase, respectively, compared to soleus controls (Fig. 1). Wada and colleagues (2014) described ~5% increase in calcium deposits in *tibialis anterior* muscle from *mdx*^20^. These results show that a high accumulation of calcium deposits may be specific to EDL from *mdx* at 12 wks.

Calcium handling by mitochondria is a key feature of eukaryotic cells: it is involved in energy production, in buffering and shaping cytosolic calcium movements and in the regulation of apoptosis^21^. Since increased calcium content was a feature of EDL *mdx* muscle at 12 wks, we decided to analyze whether the increase in calcium was related to mitochondrial function at this stage of the DMD disease.

**Figure 2:**
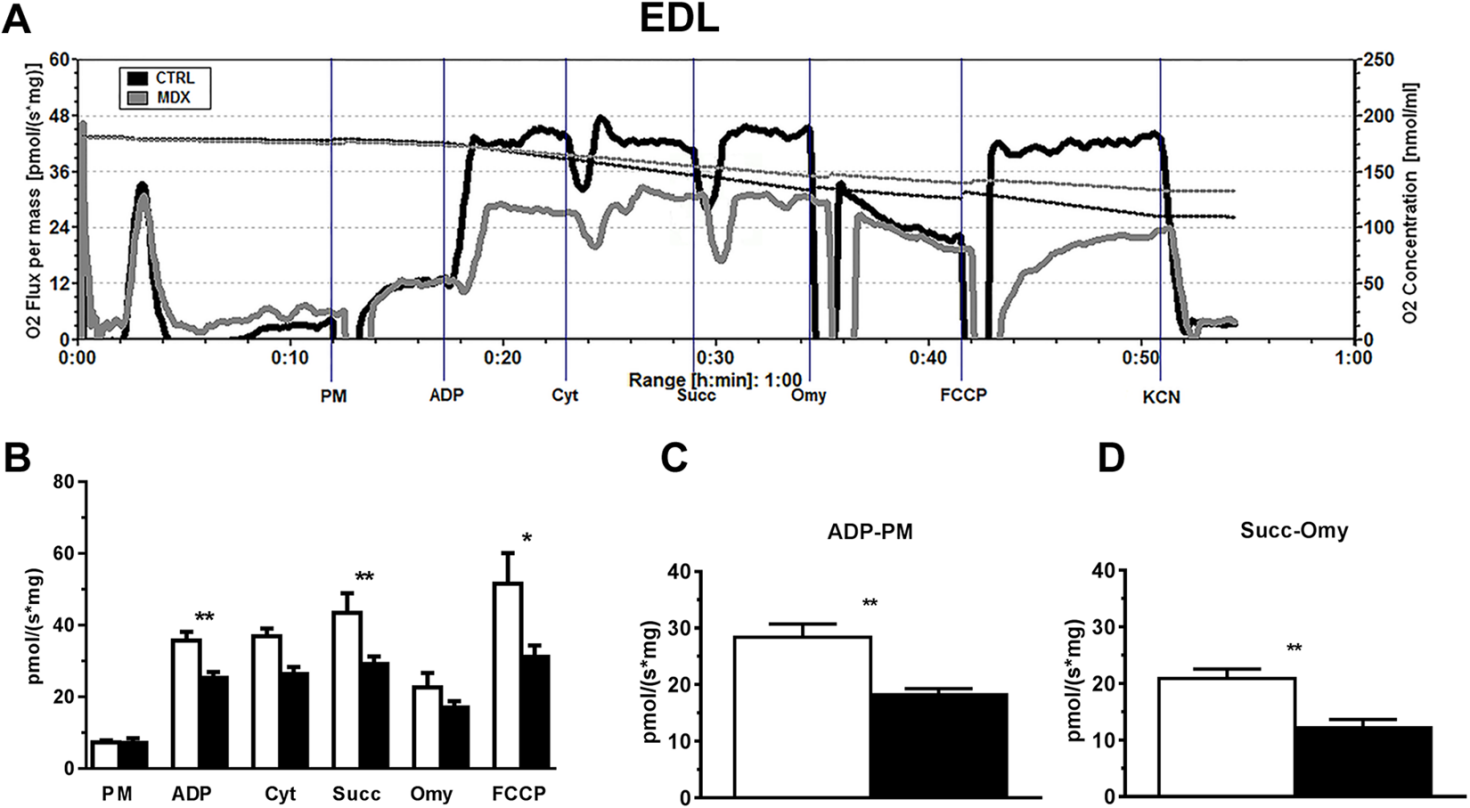
Assessment of oxygen flux in high-resolution respirometry experiments using fibers from EDL muscles of 12-wk-old control and *mdx* animals. (**A**) Representative trace of the real-time, high-resolution respirometry experiments. A multiple-substrate protocol was used to assess complex I (PM) and complex II (Succ) participation in respiration in permeabilized fibers from *extensor digitorum longus* (EDL). The additions are shown by vertical lines, with each event identified along the bottom. Pyruvate + Malate (5 mM each); ADP (3 mM); Cytochrome C (10 μM); Succinate (10 mM); Oligomycin (3 μg/mL); FCCP (1 μM); potassium cyanide (KCN, 10 mM). Control animals (black line) and *mdx* animals (gray line). The continuous lines represent the flux of O_2_ consumption [pmol/(s*mg)], and the dotted lines represent the O_2_ concentrations (nmol/mL). In (**B**–**D**) the white bars represent control and black bars represent *mdx*. (**B**) O_2_ consumption rates in high-resolution respirometry obtained from permeabilized fibers of control and *mdx* mice run in parallel. ADP **p = 0.0030, Succ **p = 0.0070, FCCP *p = 0.0379 compared to the indicated group. The graph shown in (**C**) corresponds to subtraction of the oxygen flux observed after ADP addition and that measured only with PM, **p = 0.0011; The graph shown in (**D**) corresponds to subtraction of the oxygen flux observed after oligomycin administration from that measured after Succ addition, **p = 0.0047. The data are expressed as the mean ± SD of 9 animals per experimental group.

To assess mitochondrial function, we isolated fibers from EDL and soleus muscles from 12 wks *mdx* dystrophic and control mice and analyzed mitochondrial respiration in permeabilized fibers using commonly used substrates. Substrate combinations of pyruvate/malate (PM) were used to promote reduction of NAD by dehydrogenases to donate electrons to Complex I. In this condition, Complex II (CII) does not contribute to the oxygen consumption observed. Addition of succinate was used to assess complex II by supporting electron flux through CII via flavin adenine dinucleotide (FADH_2_). The peak that appears prior to the addition of PM in Fig. 2A is an artifact of the electrode measurement. Such alterations are particularly common near the beginning of an experiment, when the electrode is stabilizing the signal after the closure of the oxygraph chamber. The oxygen consumption by EDL muscle fibers from control and *mdx* mice is shown in Fig. 2. Basically, there was no oxygen consumption before the addition of substrate. Oxygen flux increased after the addition of pyruvate/malate (PM) but no difference was detected on comparing permeabilized EDL fibers derived from control and *mdx* animals (Fig. 2A,B). As expected, ADP addition promoted an increase in oxygen consumption and allowed us to assess the coupling of the electron transport system (ETS) to ATP synthesis and oxygen consumption. Interestingly, oxygen consumption stimulated by ADP addition was significantly diminished (29% less) in fibers from *mdx* mice compared with controls (Fig. 2A,B). In coupled mitochondria, there is a direct relationship between oxygen consumption and ADP phosphorylation^22^. These results suggest that oxygen consumption stimulated by ADP addition is compromised in the EDL from *mdx* animals and may be associated with observations of loss of muscle function at this stage of the disease (12 wks). However, these results could also occur if there were mitochondrial membrane damage during the preparation of muscle fibers. To rule out this possibility, we added cytochrome c to verify the integrity of the external mitochondrial membrane^23^. In case of a mitochondrial membrane leak, the addition of cytochrome c would promote an increase in oxygen consumption. However, addition of cytochrome c after ADP caused no change, confirming the viability of the preparations (mitochondrial membrane integrity) during the experiments (Fig. 2A,B). Furthermore, after succinate was included in the reaction medium, we observed a small increase in the oxygen flux (18% and 10% in control and *mdx* group, respectively), but the differences between animal groups (control vs *mdx*) were maintained (Fig. 2A,B). The oxygen consumption in permeabilized EDL fibers derived from *mdx* animals was 33% lower than in those from control animals after succinate addition. The inclusion of oligomycin in the reaction medium decreased the mitochondrial oxygen consumption rate, but control was still higher than *mdx* fibers. The uncoupled state elicited by FCCP addition allowed us to compare the maximal respiratory capacity and confirm the lower response of fibers derived from *mdx* mice. These results show a decrease in mitochondrial function in EDL muscle from *mdx* at 12 wks.

Next, we investigated whether the changes observed in the fast-twitch muscle tissue were also observed in slow-twitch muscles. We repeated the oxygen consumption experiment using permeabilized fibers from the slow-twitch muscle soleus of control and *mdx* animals at the same age (12 wks).

**Figure 3:**
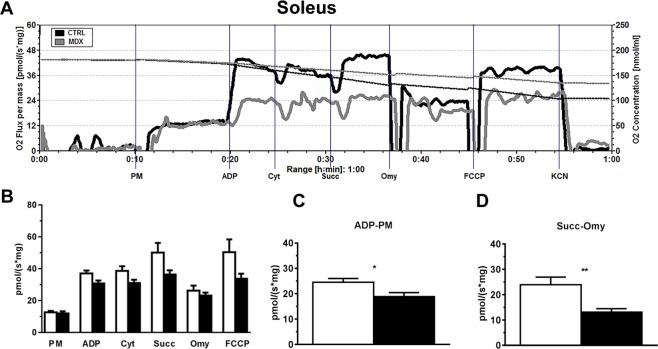
Assessment of oxygen flux in high-resolution respirometry experiments using fibers from soleus muscle of 12-wk-old control and *mdx* animals. (**A**) Representative trace of the real-time, high-resolution respirometry experiments. A multiple substrate protocol was used to assess complex I (PM) and complex II (Succ) participation in respiration by permeabilized fibers from soleus muscle. The additions are shown by vertical lines, with each event identified along the bottom. Pyruvate + Malate (5 mM each); ADP (3 mM); Cytochrome C (10 μM); Succinate (10 mM); Oligomycin (3 μg/mL); FCCP (1 μM); potassium cyanide (KCN, 10 mM). Control animals (black line) and *mdx* animals (gray line). The continuous lines represent the flux of O_2_ consumption [pmol/(s*mg)], and the dotted lines represent the O_2_ concentrations (nmol/mL). In (**B**-**D**) the white bars represent control and black bars represent *mdx*. (**B**) O_2_ consumption rates in high-resolution respirometry obtained from permeabilized fibers of control and *mdx* mice run in parallel. ADP (p = 0.075), Succinate (p = 0.1672) and FCCP (p = 0.0927) comparing *mdx* with control group. The graph shown in (**C**) corresponds to subtraction of the oxygen flux observed only with PM from that measured after ADP addition, *p = 0.0206. The graph shown in (**D**) corresponds to subtraction of the oxygen flux observed after oligomycin administration from that observed after Succ addition, **p = 0.0079. The data are expressed as the mean ± SD of 9 animals per experimental group.

As shown in Fig. 3, fibers derived from soleus had a very similar profile compared to fibers from EDL muscle. Importantly, no significant differences in mitochondrial respiration were noted between soleus fibers from *mdx* and control animals (Fig. 3A,B). However, similarly to the experiment performed with permeabilized EDL fibers, the data show a slight decline in mitochondrial respiration after addition of ADP, succinate and FCCP (Fig. 3A,B) in the fibers isolated from *mdx* animals [ADP (p = 0.075), Succinate (p = 0.1672) and FCCP (p = 0.0927) comparing *mdx* with control group].

To better identify the differences in mitochondrial function between the two types of muscle (EDL and soleus) at 12 wks, we focused on the oxygen consumption associated with ADP phosphorylation. The results (Figs 2C and 3C) show the values obtained by subtracting the flux measured only with mitochondrial substrates for complex I from the data observed after ADP addition. There was a clear decrease in the ADP phosphorylation capacity in *mdx*-derived fibers. These data were confirmed by analyzing the values of oxygen flux obtained before and after oligomycin addition (Figs 2D and 3D). The subtraction result represents the oxygen consumption coupled to ADP phosphorylation. As observed in EDL (Fig. 2D) and soleus muscles (Fig. 3D) the coupled respiration was reduced by about 45% in the fibers derived from *mdx* animals.

**Figure 4:**
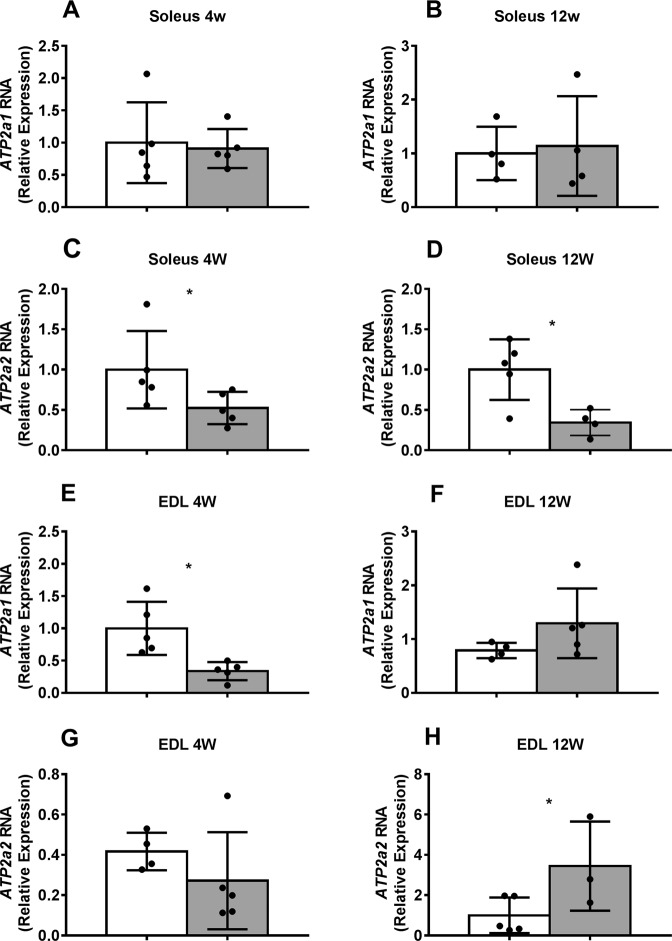
Expression of *Atp2a1* (SERCA1) and *Atp2a2* (SERCA2a) mRNA in EDL and soleus muscles from 4- and 12-week-old *mdx* and C57 control mice. Total RNA was extracted from muscles for qPCR analysis of the expression of *Atp2a1* (SERCA1) and *Atp2a2* (SERCA2a). Soleus (**A**–**D**) and EDL (**E**–**H**) muscle *Atp2a1* (SERCA1 - **A**,**B**,**E**,**F**) and *Atp2a2* (SERCA2a - **C**,**D**,**G**,**H**) mRNA expression from 4- and 12-week-old *mdx* (grey bars) and C57 control mice (white bars). N = 3–5 qPCR. Values expressed as mean ± SD. *p < 0.05.

Since we found a decrease in mitochondrial function together with an increase in calcium deposits in EDL and soleus muscles from *mdx*, we decided to analyze whether these changes could be correlated with alterations in the expression of sarcoendoplasmic reticulum (SR) calcium transport ATPases (SERCAs). SERCAs are pumps that transport calcium ions from the cytoplasm into the SR^24^ and therefore could be related to the increased calcium found in *mdx* muscles. We analyzed the expression of *Atp2a1* (SERCA1) and *Atp2a2* (SERCA2a) mRNAs in EDL and soleus muscles from 4- and 12-wks *mdx* dystrophic and C57 control mice using real-time PCR (qPCR). There was no significant difference between control and *mdx* soleus muscle in *Atp2a1* (SERCA1) mRNA levels from either 4- or 12-wks mice, nor in EDL muscle from 12-wks mice (Fig. 4A,B,F). However, expression of SERCA1 in EDL from 4-wks *mdx* mice was significantly lower than the control (Fig. 4E). In addition, there was a significant decrease in the expression of *Atp2a2* (SERCA2a) mRNA levels in soleus muscle from both 4- and 12-wks *mdx* (Fig. 4C,D) and an increase in SERCA2a mRNA from EDL of *mdx* muscle at 12 wks (Fig. 4H); and no significant differences were found in EDL of *mdx* muscle at 4 wks (Fig. 4G). These results show alterations in the expression of *Atp2a1* (SERCA1) and *Atp2a2* (SERCA2a) mRNAs from *mdx* EDL and soleus muscles.

## Discussion

Duchenne muscular dystrophy (DMD) is the most common and lethal form of human muscle disease^25^. Even with intense ongoing research, there is yet no known cure or effective treatment for DMD. The *mdx* mouse with defective dystrophin expression is one of the most widely used animal models for DMD research^5,6^, but different from human DMD, *mdx* adults (12 wks) present a mild phenotype and a less severe disease course. Despite histological signs of muscle degeneration, necrosis and inflammation, most of the body muscles are efficiently regenerated in adult *mdx* mice^5,6,11^. Therefore, understanding the mechanisms by which 12-wk-old *mdx* muscles can efficiently regenerate, while human DMD muscles cannot, is of special importance in this field and can open new possibilities for DMD treatment and therapy.

Here, we focused our efforts on the study of mitochondrial function and its possible correlation with calcium deposits and SERCA expression in slow- and fast-twitch muscles from *mdx* mice at 12 wks.

Taken together, our results show decreased mitochondrial function (respiration), increased calcium deposits and increased *Atp2a2* (SERCA2a) RNA expression in fast-twitch muscle (EDL) in the *mdx* mice at 12 wks. Conversely, slow-twitch muscle (soleus) from *mdx* mice at 12 wks did not show significant differences in mitochondrial function, presented more modest calcium deposits compared to *mdx* EDL muscle, and showed decreased *Atp2a2* (SERCA2a) RNA expression when compared to control soleus muscle (12 wks). It is important to point out that despite the lack of alterations in mitochondrial respiration, a decrease in oxygen consumption associated with ADP phosphorylation capacity was found in *mdx*-derived fibers from soleus (12 wks).

The drugs and substrates that we used in High-Resolution Respirometry, and in the sequence that they were employed, allowed an overview of the most important mitochondrial parameters in respiration: respiration with substrates and ADP, coupled respiration fraction, proton leak respiration fraction and maximal respiration. In our study we used: (i) ADP and oligomycin, which interfere with ATP synthase/complex V activity; (ii) factors that directly interfere with the membrane potential, such as FCCP; and (iii) pyruvate + malate and succinate, which are substrates for complexes I and II, respectively. Complex I and complex II are commonly the main ports of entry for electrons in the mitochondrial respiratory chain and interruptions in their supply usually lead the oxygen consumption to zero. Although we focus our studies on complexes I and II, alterations in their function have a direct impact on the remaining members of the respiratory chain. Changes in ADP phosphorylation lead to alterations in the mitochondrial membrane potential, and as a result cause a reduction in the whole chain activity.

This is the first description of mitochondrial respiration in muscles from 12-wk-old *mdx* mice using multiple mitochondrial function parameters for the comparison of EDL (fast-twitch muscle) and soleus (slow-twitch muscle). It is important to point out that even with a pronounced reduction in mitochondrial respiration, fast-twitch muscles (EDL) from 12-wk-old *mdx* mice undergo a regeneration process^11^. One possible explanation comes from previous work showing that satellite cells from dystrophic *mdx* mice display accelerated differentiation^26^, and this process may overcome the *mdx* deficiency in mitochondrial function.

Different muscles from *mdx* mice present characteristic ultrastructure, physiology and pathology^27^, partly dependent upon fiber composition (slow- or fast-twitch fibers). EDL is a homogenous fast-contracting muscle and soleus is a slow-contracting fatigue-resistant muscle. Mouse muscles can present different physiologies depending on their specific anatomic location^28^: in the limbs, anterior muscles (EDL) are faster than posterior muscles (soleus). Fast-twitch muscles from dystrophic *mdx* mice are especially susceptible to contraction-induced injury^29,30^, which could contribute to an increased calcium influx in EDL from *mdx*. This may explain our results showing that the fast-twitch muscle EDL is more affected (than soleus) in *mdx*, with increased calcium deposits and reduced mitochondrial respiration. High calcium concentrations are necessary for the activation of proteases, such as calpains, thus suggesting that fast-twitch fibers (EDL) are more susceptible to calcium-dependent proteolysis than slow-twitch fibers (soleus).

It has been shown by different groups that mitochondria play a role in DMD^9,10,14–19^. Calcium entry owing to sarcolemmal tears caused by the fragility of the dystrophin-deficient sarcolemma and by the calcium leak channels results in calcium overload in the dystrophic muscle^17^. Mitochondria serve as a buffer for part of this calcium overload. This causes a decline in mitochondrial respiratory function which in turn affects muscle function. Interestingly, it has been shown that sarcolemmal injury causes mitochondria to cluster at the site of muscle repair^31^.

We also analyzed the expression of *Atp2a1* (SERCA1) and *Atp2a2* (SERCA2a) mRNAs in EDL and soleus muscles from control and *mdx* animals, aiming to correlate it with mitochondrial function and calcium deposits. It has been reported that DMD muscles present alterations in SERCA activity^32–34^, associated with higher cytoplasmic calcium levels and alterations in mitochondrial permeability transition pore formation^35,36^. Here we found an increase in *Atp2a2* (SERCA2a) mRNA levels in EDL muscle from 12-wk *mdx* compared to age-matched controls, which may be correlated with the decrease in mitochondrial function and the increase in calcium deposits found in this muscle. While both EDL and soleus from *mdx* at 12 wks showed calcium deposits (compared to control), we found 3-fold more deposits in EDL than in soleus. We hypothesize that the abnormal calcium overload found in EDL muscles from *mdx* mice leads to an increase in SERCA2a expression to reestablish normal cytoplasmic calcium levels, since SERCA pumps transport calcium ions from the cytoplasm into the SR. In agreement with our results, Schertzer and colleagues (2008) found that transcript levels of SERCA2a are higher in EDL muscles from 20-week-old *mdx* mice compared with C57 control mice^37^.

Interestingly, a decrease in *Atp2a1* (SERCA1) RNA expression was found in fast-twitch muscle (EDL) during the myonecrosis stage in the *mdx* mice (4 wks) compared to control, whereas no significant differences were found in its expression in EDL during the regeneration stage (12 wks). These results suggest a recovery of the expression of SERCA1, the main SERCA found in EDL muscle, during the regeneration stage of the disease.

In contrast with the observed increase in the expression of *Atp2a2* (SERCA2a) in EDL from *mdx* at 12 wks (compared to control), there was a decrease in the expression of *Atp2a2* (SERCA2a) at 12 wks in soleus muscles from *mdx* animals (compared to control). Furthermore, we found a significant decrease in the expression of *Atp2a1* (SERCA1) in EDL from *mdx* mice at 4 wks, compared to the control at 4 wks; while no differences were detected in SERCA1 expression in soleus from *mdx* mice at 4 wks in relation to control. Since slow-twitch muscles (soleus) from dystrophic *mdx* mice are not especially susceptible to contraction-induced injury^29,30^ compared to fast-twitch muscles (EDL), our data suggest that the expression of SERCA1 and SERCA2a could be modulated in *mdx* muscles by the levels of calcium deposits and contraction-induced injuries. Furthermore, type I muscle fibers are less sensitive to the *mdx* injury^29,30^, thus the increase in SERCA2a expression in EDL from *mdx* mice at 12 wks could be associated with the regeneration process observed in the *mdx* mice at this age^14^.

It has been shown that different DMD muscles have different abilities to regulate calcium homeostasis^38^, and they also differ in mechanical properties that can offer resistance to damage^39^. Fast-twitch muscles are generally the most susceptible to muscular dystrophy and ageing in all species^40^. Our data are in accordance with the concept that the fast-twitch muscle EDL from *mdx* is more sensitive to alterations in calcium homeostasis than the slow-twitch muscle soleus.

Our work suggests a role for calcium in the regulation of mitochondrial respiration. It has been reported that increases in cytoplasmic calcium result in changes in the activity of mitochondrial dehydrogenases, such as FAD-glycerol phosphate dehydrogenase, pyruvate dehydrogenase phosphatase, NAD^+^-isocitrate dehydrogenase and 2-oxoglutarate dehydrogenase^41^. The activity of mitochondrial dehydrogenases drives the respiratory chain and ATP synthesis.

Taken together, our data show that EDL from *mdx* mice at 12 wks is altered to a greater extent than soleus from *mdx* mice at 12 wks. These alterations include: increased calcium deposits, reduced mitochondrial activity, and increased expression of both SERCA1 and SERCA2a. These results show that fast-twitch muscles from *mdx* are more susceptible to injury than slow-twitch muscles.

Although our study did not answer the question of why 12-wk *mdx* muscles can efficiently regenerate while human DMD muscles cannot, we did find a decrease in the overall mitochondrial function in 12-wk *mdx* muscle, which is in contrast with data from human DMD muscles. A significantly higher activity of mitochondrial complex I was observed in patients with DMD^42^. The difference in the activity of complex I in DMD muscles from humans and mice could be related to the differences in their regenerative capacities. More studies are necessary to sort out the exact role of complex I in DMD pathophysiology in humans and in different animal models.

Our results show important physiological differences between fast (EDL) and slow-twitch (soleus) muscles from 12-wk-old *mdx* mice. Our data could have an important impact on the understanding of the pathophysiology of DMD.

## Methods

### Ethics statement

This study followed the principles of good laboratory animal care and experimentation in compliance with ethical recommendations in the guidelines of the Brazilian College for Animal Experimentation. The study protocol for handling of animals was approved (protocol number: CEPA 00174/09) by the Ethics Committee for Animal Research (Comitê de Ética em Pesquisa Animal, CEPA) of the Universidade Federal Fluminense (UFF, Niterói, Rio de Janeiro, Brazil).

### Animal and muscle handling

Isogenic male 4 and 12 week-old-mice *mdx* dystrophic and age-matched C57BL/10J (C57) control non-dystrophic mice were kept in the animal housing facilities at the Institute of Biology in the Universidade Federal Fluminense (Rio de Janeiro, Brazil). Mice were housed in a ventilated rack (Alesco, São Paulo, Brazil) in autoclaved cages and kept at a constant 12 h/12 h light-dark cycle and temperature (24 °C) with free access to food and water. Mice were sacrificed by cervical dislocation and *extensor digitorum longus* (EDL) and soleus skeletal muscles were collected.

### Alizarin Red staining

At least 4 pieces of soleus and EDL muscles from each mouse were fixed in 10% neutral buffered formalin and further processed into paraffin-embedded cassettes. Sections of 5 μm in thickness were placed on poly L-lysine-coated slides and stained with Alizarin Red S 2% (ARS) solution for 3 to 5 minutes. Then, sections were washed 5 times with acetone, acetone-xylol (1:1), and xylol, for 20 seconds each step and mounted in glycerol^43^. Images of calcium deposits birefringence were observed and acquired under bright-field and polarization microscopy on an Axiovert 100 microscope (Zeiss, Germany). Images of calcium deposits were acquired under polarized light microscopy since this contrast-enhancing technique improves the quality of the image obtained with birefringent materials such as calcium deposits. Quantification of the area occupied by calcium deposits from Alizarin Red S staining was performed using Image J software.

### Acquisition of permeabilized skeletal muscle fibers

Nine independent experiments were carried out (one permeabilized fiber bundle for each group) with *mdx* dystrophic and age-matched C57 control non-dystrophic mice at 12 wks. Animals were subjected to euthanasia and EDL or soleus muscles from each group were harvested. Fiber bundles (~2 mg) were isolated in ice-cold BIOPS buffer (containing 2.77 mM CaK_2_EGTA, 7.23 mM K_2_EGTA, 20 mM imidazole, 20 mM taurine, 6.56 mM MgCl_2_, 5.77 mM ATP, 15 mM phosphocreatine, 0.5 mM dithiothreitol, and 50 mM K-MES, pH 7.1) and permeabilized using saponin at 50 µg/mL according to an established protocol^44^. After 30 min of saponin exposure, muscle fibers were washed for 10 min in respiration medium (see details under oxygen consumption measurement) and then used for respirometry analysis.

### Oxygen consumption measurement

O_2_ consumption experiments were conducted using a high-resolution Oxygraph-2k system (Oroboros Instruments GmbH, Innsbruck, Austria)^23^. The composition of respiration medium (MiR05) was: 110 mM sucrose, 60 mM K-MES, 0.5 mM EGTA, 3 mM MgCl_2_, 20 mM taurine, 10 mM KH_2_PO_4_, 20 mM K-HEPES, pH 7.1^45^. Fibers were placed in the oxygraph chamber containing 2 mL of respiration medium and experiments were performed at 37 °C. Sequential addition of substrates and drugs to the indicated final concentrations was as follows: pyruvate/malate (5 mM/5 mM), ADP (3 mM), cytochrome c (10 µM), succinate (10 mM), oligomycin (3 μg/mL), carbonyl cyanide-4-(trifluoromethoxy)phenylhydrazone) (FCCP, 0.6–1 μM) and potassium cyanide (KCN, 10 mM). Oxygen consumption was allowed to stabilize before proceeding to a new substrate/drug injection. The software Datlab5 (Oroboros Instruments GmbH, Innsbruck, Austria) was used to extract specific values of oxygen flux from chosen intervals (the areas corresponding for the addition of substrates or drugs) to plot the oxygen consumption data.

### qPCR

For the analysis of the expression of *Atp2a1* (SERCA1) and *Atp2a2* (SERCA2a) mRNA in EDL and soleus muscles from 4 and 12-week-old *mdx* and C57 control mice, total RNA was extracted using the TRIzol Reagent (Invitrogen, Carlsbad, USA) and Machery Nagel kit (Macherey Nagel, Düren, DE) according to manufacturers’ protocols. Briefly, the aqueous phase from the initial TRIzol protocol was transferred into the Machery Nagel blue column and followed downstream as in the manufacturer’s protocols. The cDNA synthesis was performed using the High Capacity cDNA Reverse Transcription Kit (Applied Biosystems, CA, USA) according to the manufacturer’s protocols with 750 ng total RNA for soleus and EDL. Followed cDNA synthesis, mRNA expressions were evaluated by qPCR using the HOT FIREPol® Evagreen® qPCR Supermix (Solis Biodyne, Denmark) in the Master Cycler Realplex system (Eppendorf, Germany). Primer pair sequences are shown in Table 1. Quantification of the samples’ mRNA expression was calculated from the standard curve method and their expression corrected by the geometric mean of the reference genes (for soleus: *Rpl0*, *Pol2a*, *Ppib*; for EDL: *Rpl0*, *G6pdh*, *Hprt1*). The best reference gene combinations were chosen according to their Cq values, using the smallest variance between the groups. The PCR program was as follows: denaturation 12 min 95 °C, 40 cycles of 15 sec 95 °C, 30 sec 60 °C, 30 sec 72 °C, following melting program. Quality of qPCR and genomic DNA contamination were checked using intron-spanning primers, reverse transcriptase-negative samples from cDNA synthesis and melting curve analysis obtained from each reaction.

**Table 1:** List of primers for qPCR.

Gene	Primer sequences	GenBank accession no.
*Ppib*	GAGACTTCACCAGGGG CTGTCTGTC TTGGTGCTCTCC	NM_011149
*Rpl0*	GGCCCTGCACTCTCGCTTTC TGCCAGGACGCGCTTGT	NM_007475.5
*Hprt1*	GCAGTACAGCCCCAAAATGG AACAAAGTCGGCCTGTATCCAA	NM_013556.2
*G6pdh*	CATGAGTCAGACAGGCTGGA GATCTGGTCCTCACGGAAAA	NM_008062.2
*Pol2a*	TCTGCCAAGAATGTGACGCT CCAAGCGGCAAAGAATGTCC	NM_001291068.1
*Atp2a1*	GGAATGCAGAGAACGCTATCG TCCTTTGCACTGACTTTCGGT	NM_007504.2 XM_006507268.1
*Atp2a2*	AATCTGACCCAGTGGCTGATG AGAGGGCTGGTAGATGTGTTG	NM_009722.3

### Statistical analysis

Statistical analyses were performed using the GraphPad prism 7 software (GraphPad Software, Inc., San Diego, CA, USA), and to assess the level of statistical significance a nonparametric Student’s t-test (Mann-Whitney test) was used with p < 0.05. The results were expressed as mean values ± standard deviation (SD). For Serca1 and Serca2a qPCR data, differences between groups were analyzed using an unpaired, one-tailed t test. Outliers were identified by Grubbs’ test and excluded. Results are shown as mean values ± SD of at least 3 animals per group.
